# Electrophysiological evaluation of cognitive intervention outcomes in children with attention deficit/hyperactivity disorder

**DOI:** 10.3389/fnins.2026.1777670

**Published:** 2026-03-31

**Authors:** Irma Khachidze, Khatuna Parkosadze, Nino Zhamureli, Ana Khachidze, Maia Advadze

**Affiliations:** 1 Laboratory of Human Psychophysiology, Ivane Beritashvili Center of Experimental Biomedicine, Tbilisi, Georgia; 2 Faculty of Medicine, Georgian National University SEU, Tbilisi, Georgia; 3 Research Institute of Cognitive Neurosciences, Free University of Tbilisi, Tbilisi, Georgia

**Keywords:** ADHD, children with ADHD, cognitive intervention, EEG, executive functions

## Abstract

**Introduction:**

Attention deficit/hyperactivity disorder (ADHD) is one of the most common neurodevelopmental disorders in children and adolescents, with an estimated global prevalence ranging from approximately 5–8%. ADHD has been associated with alterations in brain structure and function. Cognitive interventions are frequently implemented in children with ADHD, and electroencephalography (EEG) is commonly used to examine neural activity in this population. The present study describes resting-state EEG characteristics in children with ADHD before (pre) and after (post) a 3-month period of cognitive intervention, focusing exclusively on electrophysiological measures.

**Methods:**

A total of 55 children with ADHD aged 7–11 years (13 girls and 42 boys, predominantly inattentive subtype) participated in the study. Resting-state EEG was recorded pre and post a 3-month cognitive intervention. The quantitative EEG spectral analysis paradigm was performed to examine absolute power in delta, theta, alpha, and beta frequency bands, as well as the theta/beta ratio (TBR), across frontal, central, and parietal regions. All electrophysiological measures were statistically compared between pre- and post-recordings.

**Results:**

Background activity was diffuse, disorganized, and heterogeneous in both pre- and post-EEG. Resting-state EEG demonstrated elevated low-frequency activity and increased TBR across frontal, central, and parietal cortical regions, consistent with commonly reported electrophysiological patterns in ADHD. No significant pre–post differences were observed in absolute power spectra or TBR across recording conditions or cortical regions. Additionally, elevation of frontal alpha was observed under open-eyes conditions.

**Conclusion:**

Two possible explanations may account for the absence of observable changes in the typical ADHD EEG profile, characterized by elevated low-frequency power and accompanied by increased theta/beta ratio across frontal, central, and parietal regions. A 3-month observation period might not be optimal to produce changes in neurophysiological measures detectable on EEG. Ongoing developmental processes may contribute to variability in cortical organization, potentially obscuring subtle temporal changes in EEG measures. Additionally, the observed elevation of frontal alpha activity may reflect broader neurophysiological variability rather than solely delayed cortical maturation. Future research may help clarify the neurobiological links between longitudinal EEG dynamics and functional developmental trajectories to identify potential neurophysiological biomarkers in children with ADHD.

## 1 Introduction

Attention deficit/hyperactivity disorder (ADHD) is one of the most common neurodevelopmental disorders in children and adolescents, with estimated global prevalence ranging from approximately 5–8% (Ayano et al., 2023; Salari et al., 2023). ADHD is associated with difficulties in social relationships, academic achievements, and long-term health outcomes (Lawrence et al., 2021; Jennum et al., 2025; Harpin et al., 2016; Di Lorenzo et al., 2021). Consequently, there is growing interest in early recognition of children who may benefit from additional support for attentional and self-regulatory development, as this may facilitate the timely and appropriate implementation of interventions. Early detection and timely intervention may help to reduce symptom severity, facilitate prevention strategies, and contribute to improved long-term functional outcomes in individuals with ADHD (Shephard et al., 2022; Teasdale et al., 2023). In the domain of symptoms, ADHD is classified as predominantly inattentive, predominantly hyperactive–impulsive, or combined, which includes both inattentive and hyperactive–impulsive. The etiology of ADHD is multifactorial (Poddar et al., 2025; Nikolas and Burt, 2010).

ADHD, as a neurodevelopmental condition, is associated with a broad range of behavioral, cognitive, and mental health difficulties (Njardvik et al., 2025; De La Fuente et al., 2013). Therefore, the diagnosis of ADHD is based on a comprehensive clinical assessment conducted in accordance with established classification systems such as the Diagnostic and Statistical Manual of Mental Disorders (DSM-5) and the International Classification of Diseases (ICD-10) (American Psychiatric Association, 2022; World Health Organization, 2019). Current clinical guidelines from major health organizations, including the American Academy of Pediatrics (AAP) and the National Institute for Health and Care Excellence (NICE), recommend a multimodal diagnostic process that includes clinical interviews, behavioral rating scales from multiple informants (e.g., parents and teachers), developmental history, and physical or neurological examination (Wolraich et al., 2019; National Institute for Health and Care Excellence, 2018). At present, despite advancements in neuroimaging, no neurophysiological biomarker has been validated for routine clinical diagnostic use in ADHD (Wolraich et al., 2019; Parlatini et al., 2024; Cortese et al., 2023).

Similarly, current clinical guidelines recommend that treatment of ADHD be embedded within a multimodal therapeutic framework. Depending on symptom severity, functional impairment, developmental stage, and family preferences, pharmacological treatment may be combined with behavioral therapy, cognitive or psychosocial interventions, and educational support (Wolraich et al., 2019; National Institute for Health and Care Excellence, 2018).

Mounting evidence suggests that the ADHD symptom complex has been linked to alterations in brain structure and functions (Chen et al., 2025; Yu et al., 2023; Albajara Sáenz et al., 2019; Hoogman et al., 2017). It is known that brain maturation in children is not completed, and children are vulnerable to various influences (Grandjean and Landrigan, 2014). ADHD is characterized by an altered or delayed maturation pattern at any developmental stage (Shaw et al., 2007). It remains distinct during childhood, adolescence, and adulthood. The delay does not fully resolve with age (Brown et al., 2025). Therefore, it is important to employ a multimodal methodology and multivariate analysis for a comprehensive study of ADHD (Ball et al., 2019; Sokhadze et al., 2017). The combined cognitive and neurophysiological approach allows researchers to link the outcomes of various cognitive interventions with behavioral and brain functioning (Nigg and Casey, 2005); altogether can help improve both diagnosis and treatment. Cognitive interventions are thought to target the neural networks underlying attention, impulse regulation, and executive functioning (Wang et al., 2026). In ADHD, these interventions are increasingly assessed using neurophysiological tools, such as electroencephalography (EEG), including event-related potential (ERP) analyses to investigate whether behavioral improvements correspond to underlying neural changes (Gevensleben et al., 2009; Banaschewski et al., 2004).

EEG is a noninvasive, safe method for measuring the electrical activity of the human brain. This is a particularly suitable tool for studying the behavioral and neurophysiological/psychophysiological correlates of neurodevelopmental disorders to examine neural mechanisms underlying cognitive regulation in children with ADHD, thereby contributing to scientific and practical perspectives (Khachidze et al., 2021; Loo and Makeig, 2012; Lenartowicz and Loo, 2014). However, recent studies reporting EEG correlates of ADHD are inconsistent and, in some cases, ambiguous (Adamou et al., 2020). Promising experimental and clinical findings for heterogeneous neurodevelopmental conditions of ADHD are rarely applied in clinical settings, especially in guiding treatment or developing new therapies (Michelini et al., 2022). Nevertheless, the existing literature points to the importance of studies investigating the potential neurophysiological correlates of behavioral interventions in children with ADHD (Loo and Makeig, 2012; Lenartowicz and Loo, 2014).

The study aimed to evaluate the resting-state EEG characteristics in children with ADHD. The present study reports exclusively the EEG data collected before and after a 3-month period of cognitive intervention, to describe neurophysiological patterns in children with ADHD. Preliminary findings on the behavioral and neuropsychological outcomes of the cognitive intervention have been presented at several conferences (Khachidze et al., 2024, 2025a, 2026). An original manuscript is currently under review for a separate publication. Specifically, the 3-month cognitive intervention targeted possible enhancement of executive functions, including attention and concentration, which are primarily associated with the frontal lobe.

An EEG spectral analysis paradigm was then employed to assess pre- to post-intervention changes in selected EEG measures to evaluate the electroencephalographic pattern associated with ADHD. Dysregulation of background EEG activity and excessive Theta power has been frequently reported as a neurophysiological marker of ADHD (Arns et al., 2013; Markovska-Simoska and Pop-Jordanova, 2017; Barry et al., 2003). In the present study, absolute power spectra for the following four frequency bands: high delta (2–4 Hz), theta (4–8 Hz), alpha (8–13 Hz), and low beta (13–24 Hz) were carried out. Spectral analysis of each band was performed in three cortical regions: frontal, central, and parietal. The Frontal lobe (prefrontal area) is a focus for the assessment of neurophysiological changes related to attention and concentration deficits (Bach-Morrow et al., 2022; Li et al., 2025; Kopańska and Trojniak, 2025). The theta/beta ratio (TBR), particularly in fronto-central regions, has been proposed as a potential electrophysiological marker of attentional regulation in ADHD (Arns et al., 2013). High TBR is considered a potential biomarker for functional assessment of inattention and has been widely used to evaluate the effectiveness of various cognitive interventions (Arns et al., 2013; Snyder and Hall, 2006). Therefore, TBR was included in our analysis to examine intervention-related changes in cortical arousal and attentional processes. However, findings across studies remain inconsistent, and the diagnostic specificity and reliability of TBR remain debated. Methodological differences, age effects, and sample heterogeneity likely contribute to reported variability (Kiiski et al., 2020; Boxum et al., 2025). In the present study, TBR was therefore treated as an exploratory neurophysiological parameter commonly examined in ADHD research rather than as a validated diagnostic biomarker. The present study examines pre- to post-intervention changes in EEG biomarkers following a 3-month cognitive intervention to investigate neurophysiological changes in children with ADHD.

We believe that the obtained findings contribute to elucidating alterations in brain regulatory systems in children with ADHD (predominantly in the inattention subtype). The implications of this study’s findings may be significant across multiple disciplines: in pediatrics for early identification and monitoring of neurodevelopmental trajectories; in neurology for understanding brain network dysregulation; and in child and adolescent psychiatry and clinical psychology for tailoring interventions and evaluating treatment efficacy based on objective biomarkers.

## 2 Materials and methods

### 2.1 Participants

A total of 55 children diagnosed with ADHD (13 girls and 42 boys), aged 7–11 years [M = 8.6, standard deviation (SD) = 1.5], participated in the study. Children with ADHD were recruited from secondary schools and psychological centers. For all participants, ADHD diagnoses were established prior to study enrolment by a multidisciplinary team of a neurologist, a psychiatrist, a psychologist, and a pediatrician operating under the Ministry of Health. ADHD diagnosis was performed in accordance with the internationally recognized standards of DSM-5 and ICD-10 criteria. The research team did not conduct independent diagnostic reassessments and did not have direct access to detailed individual diagnostic documentation; therefore, we could not report screening details of children with ADHD. Participants were recruited from an existing cohort of school-aged children who had previously undergone formal diagnostic evaluation through the multidisciplinary assessment process.

A relatively homogeneous group of children with ADHD was enrolled in the study (predominantly inattentive) who had no comorbid diagnoses. Children with comorbid behavioral disorders, such as conduct disorder (CD), oppositional defiant disorder (ODD), or anxiety or depressive symptoms, regular pharmacological treatment, autism spectrum, mental or movement disorders, or learning disabilities were excluded from the study. None of the participating children had a history of pharmacological treatment for ADHD. The inclusion criteria were an intelligence quotient (IQ) of 80 or above. The Test of Nonverbal Intelligence, Fourth Edition (TONI-4) was administered to assess intellectual functioning. We focused on children with the predominantly inattentive subtype of ADHD, because the primary objective was to examine whether cognitive intervention can change attention. In addition, limiting the sample to this subgroup reduced clinical heterogeneity and minimized potential interfering effects of hyperactivity-related movement on resting-state EEG recordings.

The study was conducted in accordance with the ethical standards of the Declaration of Helsinki and approved by the Bioethics Committee of Ivane Beritashvili Center of Experimental Biomedicine, Tbilisi, Georgia (approval number: 02/06.04.2023). Written informed consent was obtained from the children’s parents or legal guardians. Verbal assent was obtained from the children. Participants’ anatomy was preserved, and all data were pseudonymized. Participants received age-appropriate rewards and small gifts for maintaining their motivation to participate in the research.

### 2.2 Procedure

The study examined neuropsychological and electrophysiological measures in children with ADHD over a 3-month period of cognitive intervention.

The cognitive intervention was conducted during 3 months and consisted of various types of Cognitive Computer Tests (CCTs) and Cognitive Paper Exercises (CPEs). The PEBL 2 application was used to administer computer tests, which are characterized by high reliability and validity. Exercises were adapted for the Georgian population (already translated and validated in Georgian) and used to train and develop attention and concentration skills in children with ADHD. Prior to the intervention, all participants completed a trial version of each test to ensure they fully understood the instructions.

1 CCTs were administered twice a week for 3 months. The CCT consisted of four tests that focused on improving the following aspects of attention: (a) Prostate Cancer Prevention Trial (PCPT)—selective and sustained attention; (b) SIMON—divided and focused attention; (c) Peabody Picture Vocabulary Test (PPVT)—sustained attention and vigilance; (d) VSEARCH—visual–spatial and selective attention. Participants completed each test 12 times. A total of 48 computerized training sessions were administered to each participant over the 3-month intervention period. Reaction times, percentage of correct responses, and their variability were recorded and analyzed.
2 CPE conducted daily over a 3-months focused on selective and sustained attention, concentration, and resilience, and comprised the following exercises: Test on Numbers, Test on Letters, Figure Connecting, Figure Circle, D2 and P2, Bourdon Test, and Test on 8’s. The number and complexity of symbols increased gradually in proportion to exercise performance and skill improvement. A total of 90 CPE was administered to each participant over the 3-month intervention period. Each participant performed exercises of varying numbers and levels of difficulty. Accordingly, only the tendencies of correct answer and reaction time were analyzed.

Before and after cognitive intervention, participants were evaluated using the Neuropsychological Computer Assessment Tests (NCAT) and the Neuropsychological Paper Assessment Scales (NPAS).

3 Neuropsychological assessment conducted twice before and after cognitive intervention to evaluate and determine the effectiveness and sustainability (long/short-term) of cognitive intervention. The Neuropsychological evaluation included both the Neuropsychological Computer Assessment Tests (NCAT) and the Neuropsychological Paper Assessment Scales (NPAS).
3.1 NCAT consisted of five tests that measured the following aspects of attention: selective, sustained, divided, focused attention, and alertness. The results of each test were measured by the following variables: reaction times, percentage of correct responses, and their variability were recorded and analyzed. (a) Test of Attentional Vigilance (TOAV)—measures sustained attention, selective attention, and alertness. (b) FLANKER—measures divided, focused, selective attention and motor inhibition. (c) SHIFTING ATTENTION—measures sustained and divided attention. (d) Stroop Test—measures selective attention. (e) GoNogo—measures sustained and selective attention and motor inhibition skills.
3.2 The NPAS assessment scales were administered to both teachers and parents to evaluate children’s attention-related difficulties. The instruments had been adapted for the Georgian population and demonstrated high construct validity. NPAS included four scales: National Institute for Children’s Health Quality (NICHQ) Vanderbilt Parent and Teacher scales (Salari et al., 2023), and Achenbach System of Empirically Based Assessment (ASEBA) of Child Behavior Checklist (CBCL) completed by parent, and ASEBA Teacher’s Report Form (TRF) completed by teacher (Lawrence et al., 2021). Each of these scales assesses different psychological and behavioral factors, but only attention-related factors were analyzed. Findings of cognitive training and neuropsychological evaluation are under review (*The Open Psychology Journal*).

The present study reports exclusively the EEG data collected before and after a 3-month period of cognitive intervention, to describe condition-related neurophysiological patterns in children with ADHD. Electrophysiological assessment was conducted twice—before (pre) and after (post) cognitive intervention. Here we present the preliminary analysis of EEG data. During EEG recording, the children sat on a comfortable chair in a quiet, dimly lit room. The participants’ EEG recordings were conducted at the same time of the day, in the morning, after good-night’s sleep and consisted of three consecutive resting-state conditions: resting wakefulness with eyes closed (CE1, 3 min), with eyes open (OE, 3 min), and once again with the eyes closed (CE2, 3 min); the total recording duration was 10 min. The CE1–OE–CE2 sequence was chosen to (a) assess baseline resting-state activity under both standard resting conditions; (b) capture reactivity to visual input (OE); and (c) examine the stability/reproducibility of resting EEG following visual engagement (CE2). This design allows evaluation of condition-dependent modulation while maintaining a structured resting-state protocol that is commonly employed in EEG research (Barry et al., 2007; Stam, 2005).

#### 2.2.1 EEG recording and data preprocessing

EEG recordings were performed using a multichannel electroencephalograph, “ENCEPHALAN” (Medicom MTD, SN:010247 Taganrog, Russia). EEG data were digitally recorded from 21 cup electrodes (Ag/AgCl) placed according to the international 10–20 system (Fp1, FpZ, Fp2, F7, F3, Fz, F4, F8, T3, C3, Cz, C4, T4, T5, P3, Pz, P4, T6, O1, Oz, and O_2_). The cap ground electrode (referred to as N) was placed on the forehead between Fp1, Fp2, and Fz, and the reference electrodes were placed at the left and right earlobes. The impedances were maintained below 10 kΩ for all electrodes. The input signals were filtered between 0.5 and 100 Hz, digitized at a sampling rate of 256 Hz, and resolution of 16 bits. Vertical electro-oculogram (VEOG) was recorded with two electrodes placed 1 cm above and 1 cm below the right eye.

EEG data were preprocessed using standardized procedures. “ENCEPHALAN” EEG analyzer program was used for data preprocessing and analysis. First, the recordings were visually inspected, and then they were filtered by a 2-Hz high-pass filter, a 40-Hz low-pass filter, and a 50-Hz notch filter. Next, independent component analysis (ICA) was performed to remove well-defined artifacts caused by eye and muscle movements and heart noise. Channels with excessive noise were interpolated when necessary. Finally, the EEG was manually inspected to verify artifact removal.

EEG data were segmented into 5-s epochs. Epochs exceeding predefined amplitude thresholds (>100 μV) or containing residual movement artifacts were rejected. A high level of motor activity was observed in children with ADHD, which led to some recordings being heavily contaminated with movement artifacts. To ensure the signal integrity, these datasets were systematically excluded from further analysis. Consequently, thirteen EEG recordings were rejected, leaving forty-two EEG recordings of children with ADHD for the final analysis. Participants excluded due to excessive movement artifacts did not differ descriptively from included participants in terms of age, sex distribution, or baseline clinical characteristics (Table 1). Sensitivity analysis conducted using G*Power indicated that, with the present sample size (*N* = 42), the study had 80% power (*α* = 0.05) to detect at least medium-sized within-subject effects (*f* = 0.25).

**Table 1:** Demographic data of included and excluded participants.

Variable	Included (*N* = 42)	Excluded (*N* = 13)
Age (mean ± SD)	8.7 ± 1.5	8.3 ± 1.4
Sex (M/F)	31/11	11/2
Medication use	None	None

Raw EEG data from 55 children (pre- and post-EEG) have been uploaded to the website https://eeghub.ge/. The corresponding author created the electronic EEG database. EEG data collected in our country were uploaded to the European Open Science Cloud (EOSC) for the first time in the scope of the “Horizon” grant.

The analysis included a minimum of three artifact-free epochs for each condition and each participant. A total of 10–15 fragments were analyzed for each participant. EEG analysis was performed using a quantitative EEG power spectral analysis paradigm, which includes frequency-amplitude characteristics across multiple frequency bands.

Spectral analysis of absolute EEG power using fast Fourier transform (FFT) was performed for the following four frequency bands: high delta (2–4 Hz; further referred to as delta), theta (4–8 Hz), Alpha (8–13 Hz), and low beta (13–24 Hz; further referred to as beta). The theta/beta ratio was calculated as the ratio of relative theta power (4–8 Hz) to relative beta power (13–24 Hz). Spectral analysis of each band was performed in three cortical regions: frontal (Fp1, Fp2, F3, F4, Fz, F7, and F8), central (C3, Cz, and C4), and parietal (P3, Pz, and P4).

The main attention was paid to the oscillatory EEG biomarker of ADHD – the band power ratio of Theta (4–8 Hz) to low Beta (13–24 Hz) activity in the central and fronto-central EEG sites. The frontal lobe (the prefrontal area) is a focus for the determination of neurophysiological changes associated with attention and concentration deficit. For EEG frequency analysis, the measurement of TBR in the fronto-central regions is considered a potential biomarker for functional diagnostic (inattention scores) and also serves as a tool for evaluating the effectiveness of various interventions.

### 2.3 Data analysis

Statistical analyses were performed using IBM Statistical Package for Social Sciences (SPSS) Statistics (version 26.0). A multivariate repeated-measures analysis of variance (ANOVA) was conducted to evaluate the changes in the EEG pattern after the cognitive intervention. The model included three within-subject factors: time (pre-intervention vs. post-intervention), Condition (CE1, OE, and CE2), and cortical region (frontal, central, and parietal). The dependent variables consisted of absolute power in the delta, theta, alpha, and beta frequency bands. Significant main effects and interactions were further examined using *post hoc* paired-samples *t*-tests. Bonferroni correction was applied to reduce the risk of Type I errors arising from multiple comparisons, and the significance level was set at *α* = 0.05. Bonferroni correction was applied to *post hoc* comparisons only.

## 3 Results

The resting state EEG was performed twice—before (pre-EEG) and after (post-EEG) cognitive intervention in 55 children with ADHD. Thirteen EEG recordings were rejected due to motor artifacts. Consequently, EEG recordings of forty-two children with ADHD remained for the final analysis. EEG procedure included the following conditions: resting wakefulness with eyes closed (CE1), with eyes open (OE), and with the eyes closed (CE2). The total duration was 10 min (overall background EEG). The resting EEG was recorded twice—pre- and post-cognitive intervention. The absolute power spectra (APS) for the delta, theta, alpha, and beta frequency bands were analyzed across frontal, central, and parietal regions and compared across time (pre- and post-cognitive intervention), functional conditions (CE1, OE, and CE2), and cortical regions.

### 3.1 Overall background EEG

The overall background EEG activity was diffuse, disorganized, and heterogeneous across participants in both pre- and post-EEG. A detailed description of EEG patterns in children with ADHD was reported in our previous articles and conference abstracts (Khachidze et al., 2022, 2024, 2025a,b; Maloletnev et al., 2016).

### 3.2 Absolute power spectra

**Figure 1:**
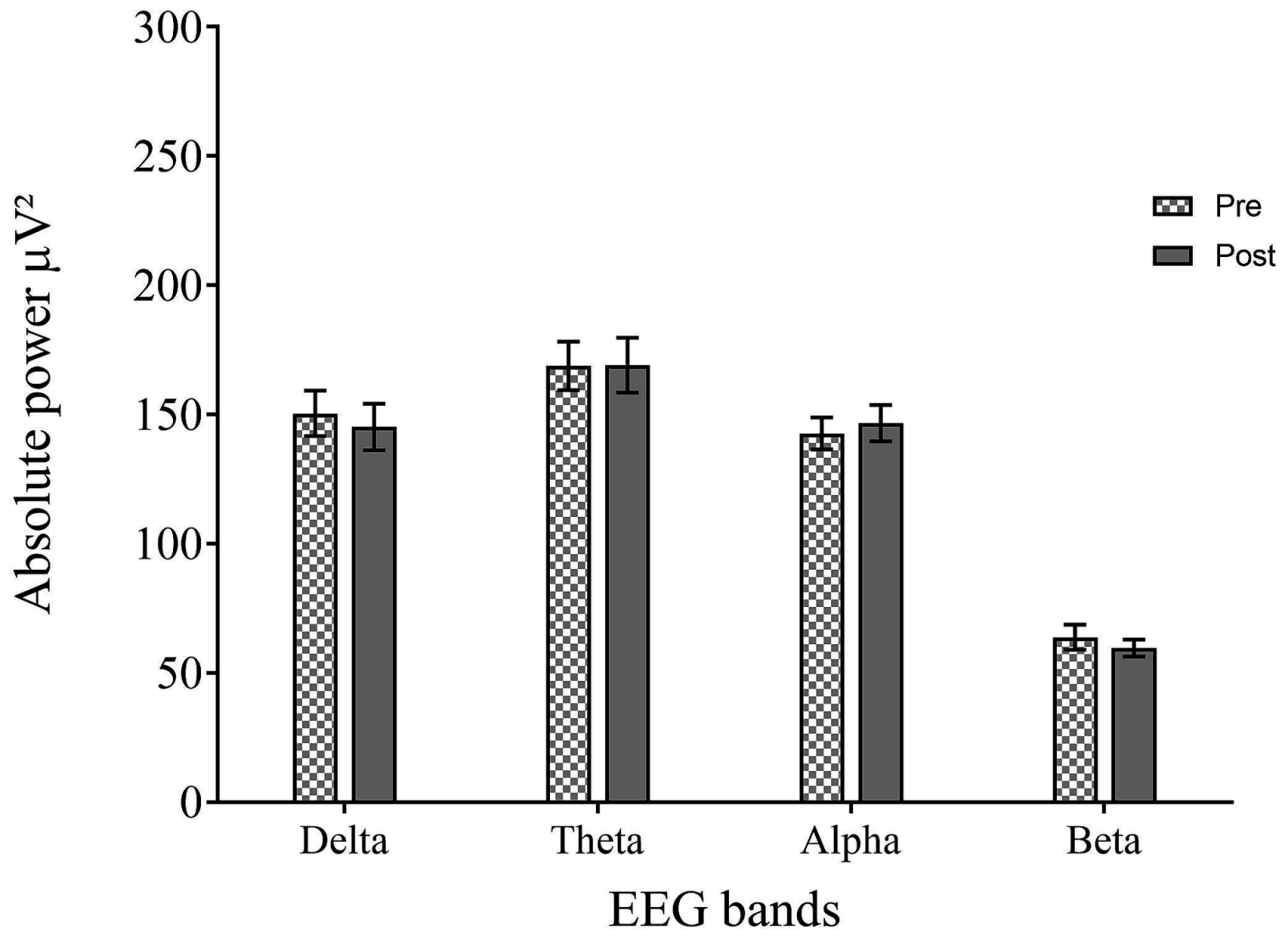
Summary of absolute power spectra (APS) in pre- and post-EEG for all frequency bands.

Pre-EEG revealed the distribution of APS in the following frequency bands: delta 26%, theta 31%, alpha 25%, and beta 12%. Post-EEG did not reveal any significant changes (*p* > 0.05 for all bands). Repeated measures ANOVA of APS of EEG bands revealed no significant differences before and after cognitive intervention for delta [*F* (1, 41) = 0.234, *p* = 0.631, *ηp*^2^ = 0.001], theta [*F* (1, 41) = 0.063, *p* = 0.803, *ηp*^2^ = 0.021], alpha [*F* (1, 41) = 2.007, *p* = 0.164, *ηp*^2^ = 0.004], and beta [*F* (1, 41) = 1.008, *p* = 0.321, *ηp*^2^ = 0.008] (Figure 1).

For delta, there was no significant main effect of the different conditions [*F* (2, 41) = 0.194, *p* = 0.825, *ηp*^2^ = 0.026], indicating a small effect size and suggesting that delta power did not differ significantly across recording conditions. However, a significant main effect was observed for cortical areas [*F* (2, 41) = 7.410, *p* = 0.002, *ηp*^2^ = 0.181], indicating a large effect size and greater delta power in frontal regions compared to other cortical areas.

For theta, a significant main effect was observed for the different conditions [*F* (2, 41) = 11.058, *p* < 0.001, *ηp*^2^ = 0.240] corresponding to a large effect size. *Post hoc* analyses indicated a significant decrease in theta power during the eyes-open condition compared to the eyes-closed condition, while no significant difference was observed between CE1 and CE2 conditions (*p* > 0.05). A significant main effect was observed in the cortical regions [*F* (2, 41) = 47.032, *p* < 0.001, *ηp*^2^ = 0.149], indicating a large effect size, with higher Theta activity in central and frontal regions.

For alpha, a significant main effect was observed for different conditions [*F* (2, 41) = 61.820, *p* < 0.001, *ηp*^2^ = 0.121], indicating a moderate-to-large effect size and reflecting a significant reduction in Alpha power in the eyes-open condition. A significant main effect was found for cortical regions [*F* (2, 41) = 92.207, *p* < 0.001, *ηp*^2^ = 0.104], indicating a moderate effect size, with higher alpha power in parietal regions.

For beta, there was a significant main effect for different conditions [*F* (2, 41) = 9.091, *p* = 0.001, *ηp*^2^ = 0.103], indicating a moderate effect size and reflecting increased beta power in the eyes-open condition compared to both eyes-closed conditions. No significant difference was found for beta between CE1 and CE2 conditions (*p* > 0.05). A significant main effect was observed in the cortical regions [*F* (2, 41) = 11.383, *p* < 0.001, *ηp*^2^ = 0.099], indicating a moderate effect size and higher beta activity in frontal regions.

**Figure 2:**
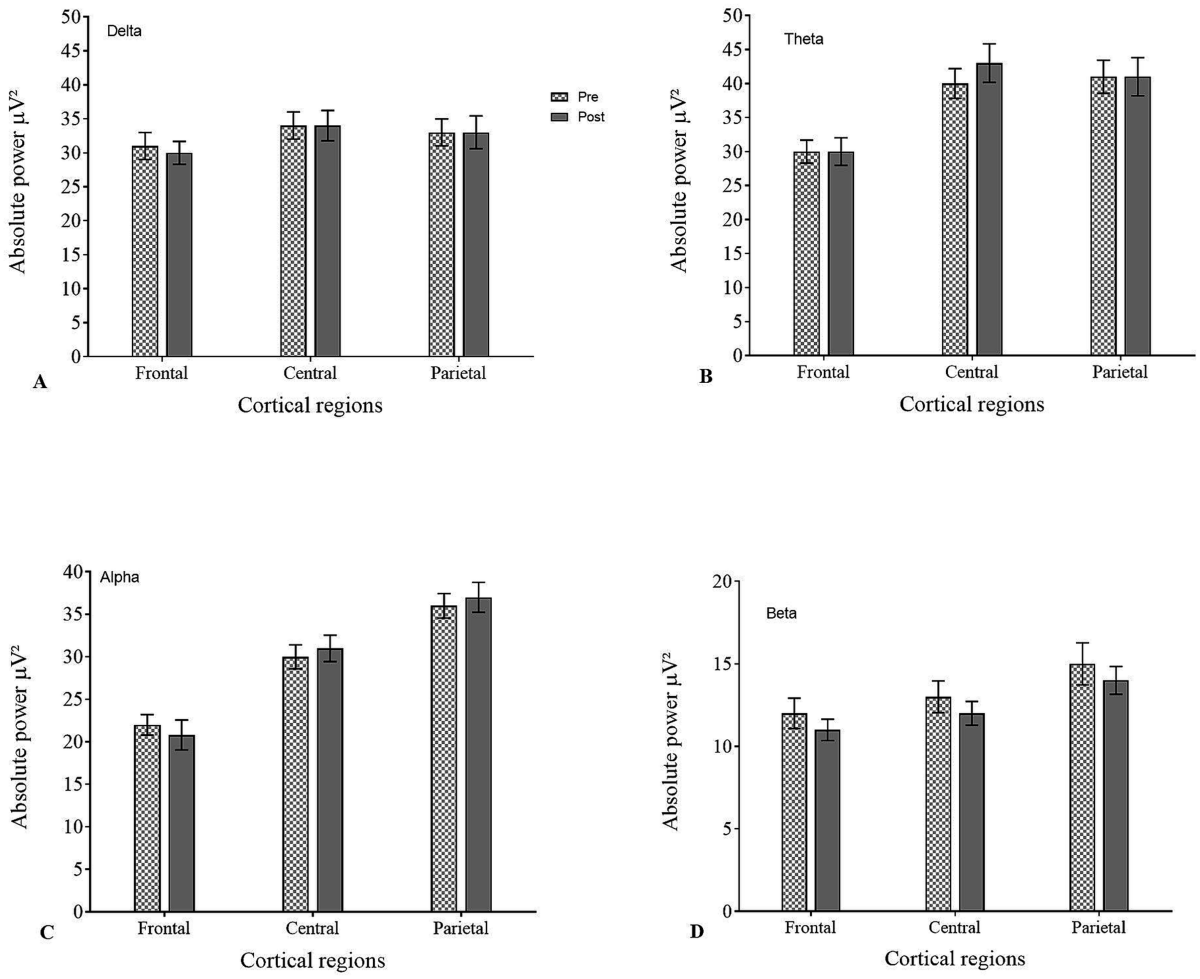
Absolute power spectra (APS) across different cortical regions in pre- and post-EEG for **(A)** delta, **(B)** theta, **(C)** alpha, and **(D)** beta.

APS analysis of pre- and post-EEG across different cortical regions for all frequency bands showed an increase in low frequencies (delta and theta) in frontal-central regions and high alpha in frontal/prefrontal regions (Table 2, Figure 2). Although the global distribution of power spectra is typical for ADHD, increased alpha power in frontal/prefrontal regions may reflect reduced cortical activation or delayed maturation.

**Table 2:** Relative distribution of EEG bands (in percent) before and after cognitive intervention, and results of paired-sample *t*-tests (*p*-values) are presented.

Condition	Frontal				Central				Parietal			
	Delta	Theta	Alpha	Beta	Delta	Theta	Alpha	Beta	Delta	Theta	Alpha	Beta
Before	29 (±6)	31 (±4)	22 (±3)	11 (±2)	27 (±5)	31 (±6)	31 (±6)	10 (±3)	25 (±4)	30 (±6)	27 (±4)	11 (±3)
After	29 (±7)	31 (±4)	21 (±2)	12 (±3)	26 (±6)	32 (±7)	32 (±6)	10 (±2)	24 (±4)	30 (±5)	28 (±5)	10 (±2)
*t*-Test (*p*)	0.663	0.081	0.148	0.124	0.717	0.306	0.198	0.117	0.285	0.418	0.809	0.086

Given the available sample size, the analyses were sufficiently powered to detect medium effect sizes; however, subtle changes of smaller magnitude may not have reached statistical significance.

### 3.3 EEG pattern during different functional conditions

#### 3.3.1 CE1 condition

**Figure 3:**
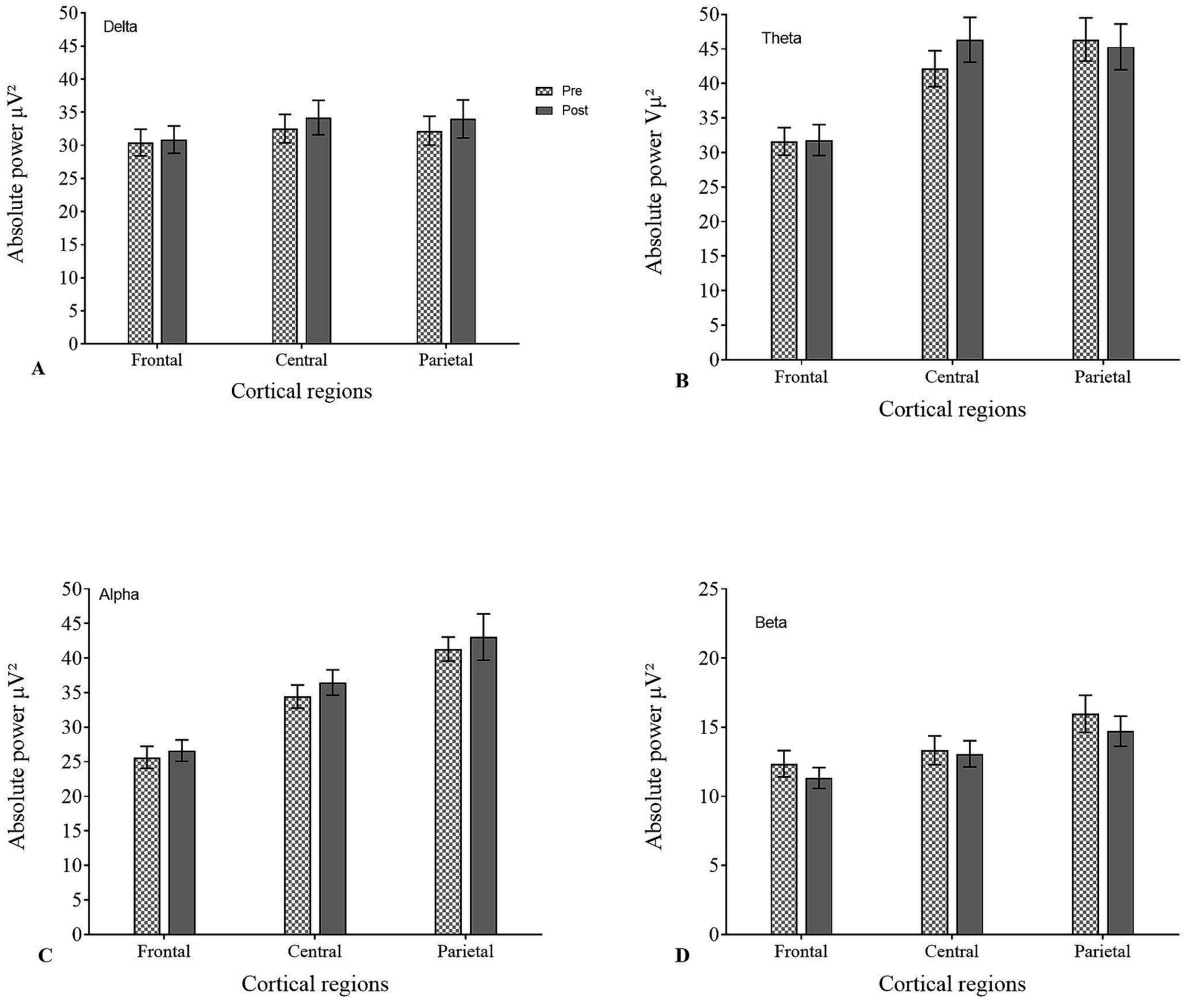
Absolute power spectra (APS) during CE1 condition across different cortical regions in pre- and post-EEG for **(A)** delta, **(B)** theta, **(C)** alpha, and **(D)** beta.

The APS analysis of pre-EEG during the CE1 condition showed the following distribution across frequency bands: delta 24%, theta 30%, alpha 27%, and beta 11%. Post-EEG analysis did not reveal any significant changes (*p* > 0.05 for all bands). The distribution of the EEG bands across cortical regions during CE1 is shown in Table 3, Figure 3.

**Table 3:** Relative distribution of EEG bands during CE1 condition (in percent) before and after cognitive intervention, and results of paired-sample *t*-tests (*p*-values) are presented.

Condition	Frontal				Central				Parietal			
	Delta	Theta	Alpha	Beta	Delta	Theta	Alpha	Beta	Delta	Theta	Alpha	Beta
Before	27 (±7)	28 (±5)	23 (±8)	11 (±4)	24 (±7)	32 (±7)	27 (±8)	10 (±3)	22 (±7)	31 (±7)	29 (±7)	11 (±4)
After	28 (±6)	28 (±6)	25 (±7)	10 (±3)	24 (±6)	32 (±6)	27 (±7)	10 (±3)	23 (±7)	30 (±7)	30 (±9)	10 (±3)
*t*-Test (*p*)	0.774	0.923	0.025	0.144	0.692	0.399	0.109	0.478	0.700	0.564	0.186	0.293

#### 3.3.2 OE condition

**Figure 4:**
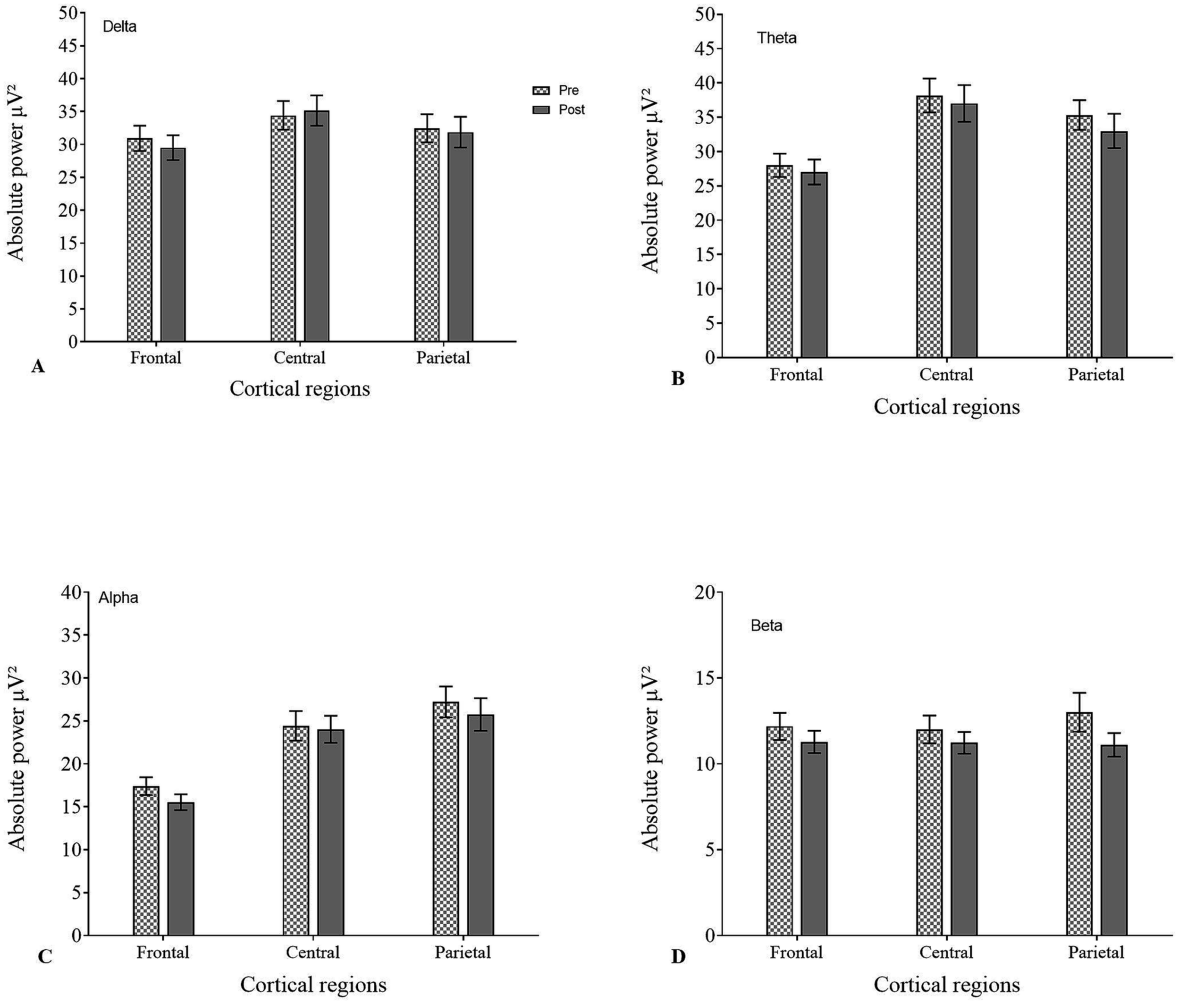
Absolute power spectra (APS) during OE condition across different cortical regions in pre- and post-EEG for **(A)** delta, **(B)** theta, **(C)** alpha, and **(D)** beta.

APS analysis of pre-EEG during OE condition showed the following distribution across frequency bands: delta 29%, theta 29%, alpha 21%, and beta 12%. Post-EEG analysis did not reveal any significant changes (*p* > 0.05 for all bands). The distribution of the EEG band across the cortical regions during OE is shown in Table 4, Figure 4.

**Table 4:** Relative distribution of EEG bands during OE condition (in percent) before and after cognitive intervention, and results of paired-sample *t*-tests (*p*-values) are presented.

Condition	Frontal				Central				Parietal			
	Delta	Theta	Alpha	Beta	Delta	Theta	Alpha	Beta	Delta	Theta	Alpha	Beta
Before	31 (±7)	28 (±5)	18 (±5)	12 (±4)	29 (±7)	32 (±5)	20 (±6)	10 (±3)	28 (±8)	30 (±6)	23 (±8)	11 (±4)
After	31 (±6)	29 (±6)	17 (±5)	12 (±3)	30 (±7)	31 (±7)	21 (±7)	10 (±3)	28 (±7)	29 (±7)	23 (±7)	10 (±3)
*t*-Test (*p*)	0.788	0.617	0.343	0.796	0.502	0.450	0.656	0.446	0.656	0.723	0.735	0.182

Power spectra of the alpha band in frontal regions on pre-EEG were decreased by 5% between CE1 (23%) and OE (18%) conditions. Power spectra of alpha band in frontal regions on post-EEG were decreased by 8% between CE1 (25%) and OE (17%) conditions (normal physiological response). Despite the decrease in Alpha between CE1 and OE conditions, this frequency remained higher in the frontal regions during the OE condition. Post-EEG did not show any significant difference (*p* > 0.05).

#### 3.3.3 CE2 condition

**Figure 5:**
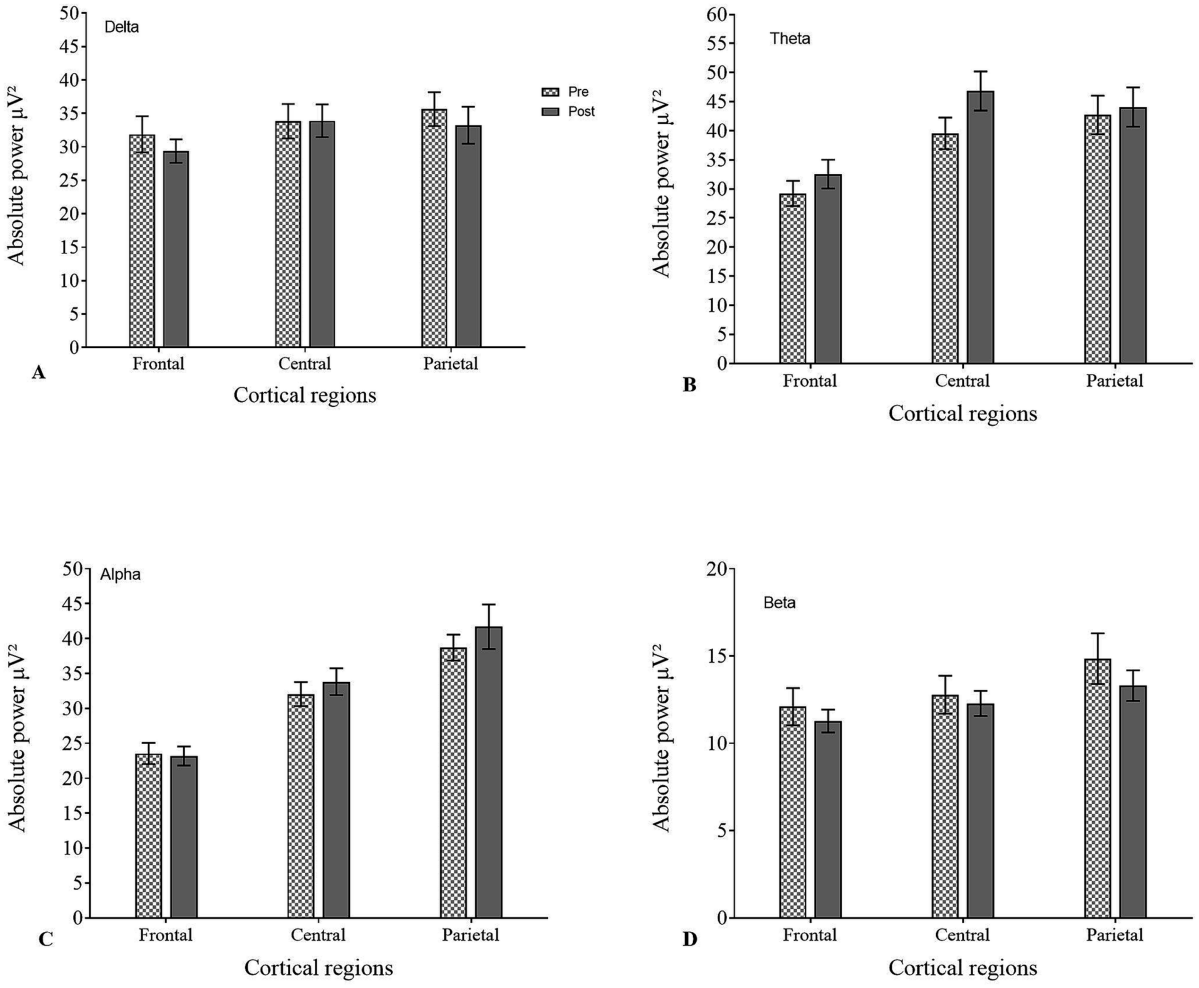
Absolute power spectra (APS) during CE2 condition across different cortical regions in pre- and post-EEG for **(A)** delta, **(B)** theta, **(C)** alpha, and **(D)** beta.

APS analysis of pre-EEG during the CE2 condition showed the following distribution across frequency bands: delta 26%, theta 28%, alpha 27%, and beta 11%. Post-EEG analysis did not reveal any significant changes (*p* > 0.05 for all bands). The distribution of the EEG bands across cortical regions during CE2 is shown in Table 5, Figure 5.

**Table 5:** Relative distribution of EEG bands during CE2 condition (in percent) before and after cognitive intervention, and results of paired-sample *t*-test (*p*-values) are presented.

Condition	Frontal				Central				Parietal			
	Delta	Theta	Alpha	Beta	Delta	Theta	Alpha	Beta	Delta	Theta	Alpha	Beta
Before	29 (±8)	27 (±6)	23 (±8)	12 (±4)	26 (±7)	31 (±7)	26 (±7)	10 (±4)	25 (±6)	29 (±6)	28 (±8)	10 (±4)
After	28 (±6)	30 (±7)	23 (±7)	11 (±3)	24 (±7)	33 (±7)	26 (±9)	9 (±3)	23 (±6)	30 (±7)	30 (±10)	10 (±3)
*t*-Test (*p*)	0.248	0.002	0.848	0.111	0.103	0.011	0.923	0.104	0.036	0.216	0.166	0.113

Across conditions, the overall distribution of EEG power spectra showed comparable patterns between CE1 and CE2 within the same recording session, with no systematic drift across frequency bands or cortical regions. This within-session consistency suggests relative stability of resting-state EEG measures under the applied recording conditions.

Some condition-specific comparisons yielded statistically significant results; however, these findings were isolated and not consistent across measures.

### 3.4 Multivariate repeated measures ANOVA for each frequency band

A multivariate repeated measures ANOVA was performed, including the following within-subject factors: time (pre- and post-EEG), conditions (CE1, OE, and CE2), and cortical regions (frontal, central, and parietal). The results of multivariate repeated measures ANOVA are summarized in Table 6.

**Table 6:** Results of multivariate repeated measures ANOVA.

EEG band	Effect	*F* (df1, df2)	*p*	*ηp* ^2^
Delta	Time	0.048 (1, 41)	0.827	0.001
	Time × condition	0.890 (2, 82)	0.414	0.021
	Time × region	0.821 (2, 82)	0.443	0.020
	Time × condition × region	0.244 (4, 164)	0.913	0.006
Theta	Time	0.809 (1, 41)	0.374	0.019
	Time × condition	2.658 (2, 82)	0.076	0.061
	Time × region	2.725 (2, 82)	0.070	0.048
	Time × condition × region	1.080 (4, 164)	0.368	0.026
Alpha	Time	0.341 (1, 41)	0.562	0.008
	Time × condition	1.509 (2, 82)	0.227	0.035
	Time × region	1.060 (2, 82)	0.351	0.025
	Time × condition × region	0.283 (4, 164)	0.889	0.007
Beta	Time	0.093 (1, 41)	0.762	0.002
	Time × condition	0.842 (2, 82)	0.473	0.015
	Time × region	0.715 (2, 82)	0.491	0.013
	Time × condition × region	0.447 (4, 164)	0.847	0.008

Across all frequency bands, no significant main effects of time were observed. For the delta band, the main effect of time was not significant, [*F* (1, 41) = 0.048, *p* = 0.827, *ηp*^2^ = 0.001], indicating a negligible effect size. Similarly, no significant time effects were found for theta, [*F* (1, 41) = 0.809, *p* = 0.374, *ηp*^2^ = 0.019]; alpha [*F* (1, 41) = 0.341, *p* = 0.562, *ηp*^2^ = 0.008]; or beta [*F* (1, 41) = 0.093, *p* = 0.762, *ηp*^2^ = 0.002].

A *post hoc* power analysis indicated that the study was adequately powered to detect medium-sized within-subject effects. However, smaller neurophysiological changes may not have been detectable given the present sample size and should therefore not be excluded.

No significant time × condition, time × region, or time × condition × region interactions were observed across frequency bands (all *p* > 0.05), with small effect sizes (*ηp*^2^ ranging from 0.006 to 0.061). Although the time × condition and time × region interactions for theta approached significance (*p* = 0.076 and *p* = 0.070, respectively), the associated effect sizes remained small (*ηp*^2^ = 0.061 and 0.048).

**Figure 6:**
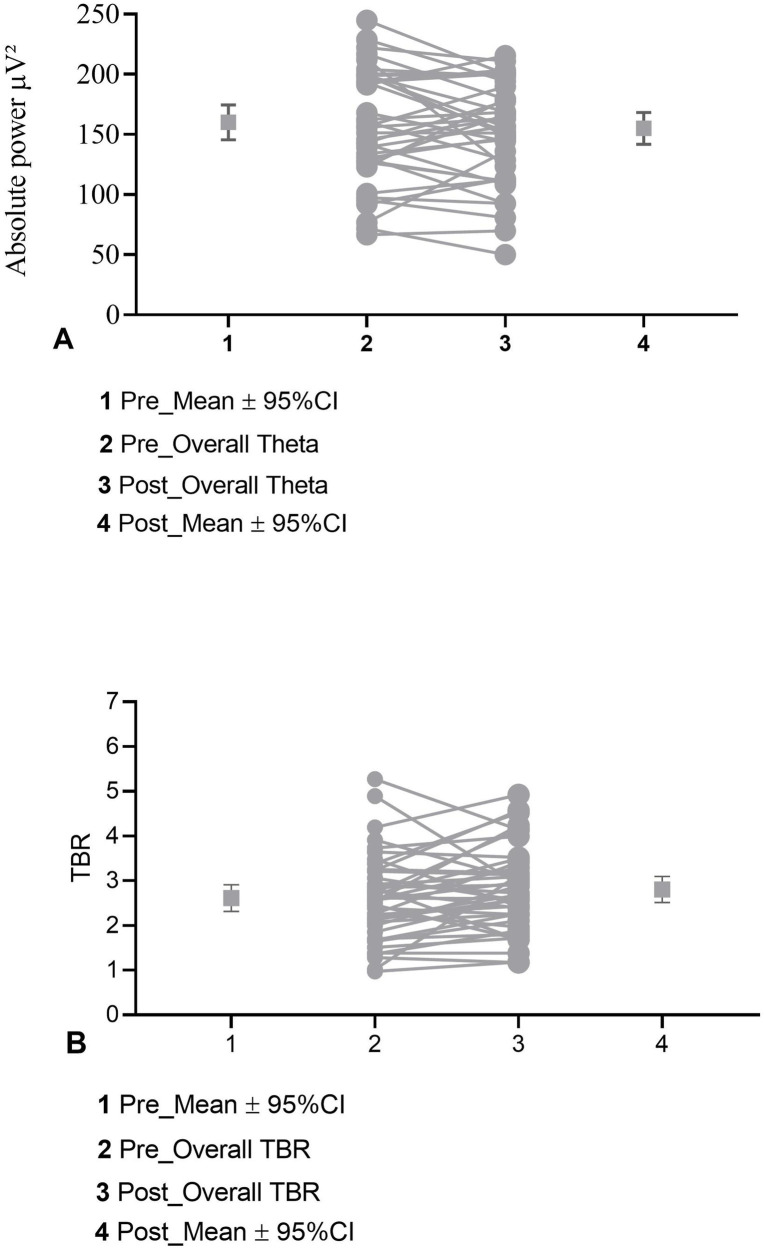
Paired pre–post plots for **(A)** overall theta, and **(B)** overall TBR.

Overall, these findings indicate minimal pre–post changes in resting-state EEG activity following the intervention. Visual inspection of paired pre–post plots further confirms the absence of systematic group-level changes (Figure 6).

### 3.5 Theta/beta ratio

**Figure 7:**
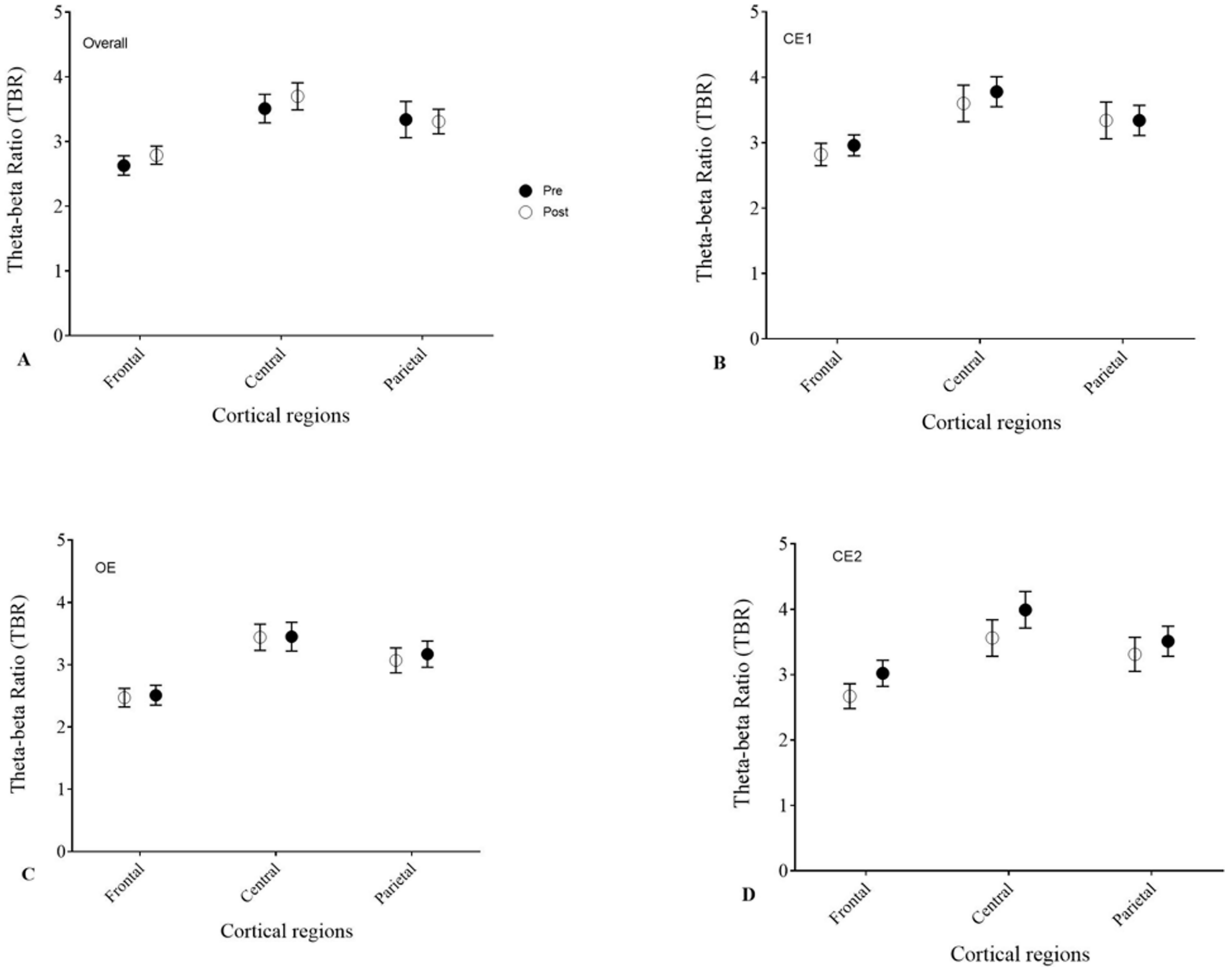
Theta-beta ratio (TBR) in pre-EEG and post-EEG across frontal, parietal and central regions during **(A)** overall background activity, **(B)** CE1, **(C)** OE and **(D)** CE2 conditions.

The theta–beta ratio (TBR) was high in the frontal, central, and parietal regions and did not change significantly between pre- and post-EEG recordings. This result is consistent with other studies that have reported elevated TBR in children with ADHD. A paired-sample *t*-test did not reveal a significant change in TBR between pre- and post-EEG (Figure 7).

While no statistically significant differences were observed, the study was powered to detect medium effects; smaller changes in TBR cannot be ruled out.

The overall distribution of power spectra revealed a typical ADHD profile (increased low-frequency bands). Specifically, our results showed that the distribution of low-frequency bands in pre-EEG was higher in frontal, central, and parietal regions: Theta was 31% in both frontal and central regions, 30% in the parietal region. Delta was 29% in the frontal region, 27% in the central region, and 25% in the parietal region. The distribution did not differ significantly in the post-EEG (*p* > 0.05). A multivariate repeated measures ANOVA showed that delta was significantly higher in frontal regions than in central and parietal regions (*p* = 0.002). There was no other significant difference for delta between conditions or pre- and post-EEG. Theta was significantly higher in frontal regions compared to central and parietal regions (*p* < 0.001). There was no other significant difference in theta between conditions or between pre- and post-EEG. The distribution of the alpha in pre-EEG was 29% in the parietal regions, 27% in the central region, and 23% in the frontal region. Multivariate repeated measures ANOVA showed that alpha was significantly higher in parietal regions compared to central and frontal regions (*p* = 0.002). There was a significant decrease in Alpha activity during the OE condition compared to the CE1 and CE2 conditions (*p* < 0.001). Despite the decrease in alpha between CE1 and OE conditions, this frequency in the frontal region remained higher over the OE condition. Multivariate repeated measures ANOVA showed that beta was significantly higher in the frontal regions compared to the central and parietal regions (*p* < 0.001). There were no other significant differences for beta between conditions and pre- and post-EEG.

TBR was high across the frontal, central, and parietal regions and did not change significantly between pre- and post-EEG recordings. Specifically, in pre-EEG, the frontal TBR was 2.8, the central TBR was 3.5, and the parietal TBR was 3.3. There was no significant difference in TBR between pre- and post-EEG.

## 4 Discussion

The study seeks to understand the neurophysiological and psychological aspects of ADHD. Various cognitive interventions were performed over a 3-months period to improve different types of attention in children with ADHD. The effectiveness of the 3-month cognitive training was evaluated using measures of brain electrical activity, that is, EEG. Specifically, we examined resting-state EEG characteristics before and after a 3-month cognitive intervention in children with ADHD. The present study focuses exclusively on electrophysiological measures; behavioral outcomes of the cognitive intervention are not addressed here.

No significant pre–post differences were observed in absolute power spectra or the theta/beta ratio across cortical regions and recording conditions. Resting-state electrophysiological markers were consistent with the typical ADHD EEG profile, showing increased theta and delta activity, and reduced beta activity. This pattern was accompanied by elevated TBR across frontal, central, and parietal regions.

The absence of significant pre–post changes in resting-state EEG in our study may reflect several alternative explanations. In contrast, the 3-month period was not long enough to elicit detectable electrophysiological changes. Our findings are in line with previous studies reporting moderate to high test–retest stability of resting EEG measures, including absolute power spectra and TBR, over short time intervals in children with ADHD (Arns et al., 2013; Clarke et al., 2001; McEvoy et al., 2000). In contrast, others reported that EEG organization typically occurs gradually over months to years rather than within short intervention periods, based on observations of neurodevelopmental changes in EEG (John et al., 1980; Marshall et al., 2002; Giertuga et al., 2017). Evidence from cognitive (attention, working memory, response inhibition, etc.) and neurofeedback interventions in ADHD showed controversial results. While some studies report improvements in behavioral and neurophysiological (EEG) outcomes following short-term training (Gevensleben et al., 2010; Geladé et al., 2018), others find limited or inconsistent effects following similar short-term training durations (Cortese et al., 2015; van Dongen-Boomsma et al., 2014; Sonuga-Barke et al., 2013). Our findings are consistent with previous reports indicating heterogeneous outcomes in short-term studies of ADHD. In the present study, no substantial changes were observed in neurophysiological measures over the 3-month period. This may reflect the gradual nature of cortical plasticity and the ongoing developmental processes of brain changes, which typically consolidate over longer periods. Consequently, EEG characteristics measured across a 3-month interval may capture intrinsic fluctuations in resting-state activity rather than changes attributable to the study period or specific interventions (Gevensleben et al., 2009; Loo and Makeig, 2012; Arns et al., 2009; Steiner et al., 2014). These observations highlight that short-term EEG measures may show partial or subtle changes, whereas longer observation periods may be required to capture more stable neurophysiological patterns. Although the sensitivity analysis indicated sufficient statistical power to detect medium-sized within-subject effects, smaller neurophysiological changes cannot be excluded. Subtle alterations in resting-state activity, if present, may therefore have remained below the detectable threshold given the current sample size and methodological approach. Future studies incorporating age-matched control groups and/or examining the neurophysiological effects of the cognitive intervention used may help to better characterize EEG variability and distinguish potential study-related changes from maturational and state-related fluctuations in children with ADHD.

High TBR observed in our findings is consistent with the results of other studies that reported increased TBR in children with ADHD (Arns et al., 2013; Luo et al., 2019). In early studies, an elevated TBR has long been considered a potential biomarker for ADHD (Barry et al., 2003; Monastra et al., 1999). Nowadays, there is evidence that TBR is not universal for all ADHD cases, and cannot be used as the only diagnostic tool (Boxum et al., 2025; Gholamali Nezhad et al., 2025). TBR is not elevated in all individuals with ADHD and is affected by other factors. TBR appears to be influenced by developmental stage, methodological factors, and sample heterogeneity. Accordingly, in the present study, TBR was interpreted as an exploratory neurophysiological parameter commonly examined in ADHD research rather than as a validated diagnostic biomarker.

According to International League Against Epilepsy (ILAE) guidelines, EEG is used for diagnostic purposes in epilepsy (International League Against Epilepsy and International Federation of Clinical Neurophysiology, 2022) and is less commonly applied in ADHD (National Institute for Health and Care Excellence, 2018; Lenartowicz and Loo, 2014; Millichap et al., 2011). It is known that the EEG pattern in ADHD reflects variability and heterogeneity (Loo and Makeig, 2012; Ebadi et al., 2025). This is confirmed by the current results and by the data presented in our previous studies (Khachidze et al., 2022, 2024, 2025a,b). Promising experimental and clinical data of heterogeneous neurodevelopmental conditions of ADHD are rarely applied in clinical settings, especially for treatment decisions and/or the development of a new approach (Michelini et al., 2022; Luo et al., 2019; Loo et al., 2024). Heterogeneity may serve as a basis for distinguishing neuropsychological, physiological, and biochemical mechanisms involved in ADHD and may reflect atypical states of the CNS (Stevens et al., 2018). Atypical brain waves and different EEG rhythms should be correlated with the function of various intracerebral features and delayed maturation in children with ADHD. In addition, different components of ADHD mature and progress in different ways—hyperactive/compulsive component matures with age, while the inattentive component remains more stable over time (Loo et al., 2018; Van Lieshout et al., 2016; Holbrook et al., 2016).

Our data showed an increase in alpha activity in the frontal region that did not change across a 3-month period. Furthermore, there was an increase in frontal alpha under the open-eyes condition. Such a pattern of EEG activity may be considered as an atypical electrophysiological activity (Clarke et al., 2001; Byeon et al., 2020). In typically developed children, at rest, Alpha activity is predominantly localized in the occipital and parietal regions (Loo and Makeig, 2012; Lenartowicz and Loo, 2014; Byeon et al., 2020; Greve et al., 2025). The frontal Alpha findings in EEG studies of ADHD are inconsistent or contradictory. In children with ADHD, Alpha power is often reduced and less focally concentrated, exhibiting a more diffuse distribution and altered frontal reactivity, suggesting atypical resting-state cortical organization (Loo and Makeig, 2012; Barry et al., 2003). These patterns reflect differences in arousal regulation rather than a simple developmental delay (Barry et al., 2003; Fonseca et al., 2013). We observed typical Alpha blocking in occipital and parietal regions, which is a standard physiological response (Barry et al., 2007; Wan et al., 2019). In addition, the frontal region showed an elevation of alpha activity during the OE condition (about 18% in the frontal region). Increased frontal alpha in children with ADHD is associated with delayed cortical maturation, which reflects persistence of inhibitory oscillatory activity in areas responsible for attentional and executive functions, and may be the sign of undiagnosed childhood depression or other mood disorders (Byeon et al., 2020; Kim et al., 2025). Other studies emphasize elevated frontal alpha as heterogeneity in cortical activation patterns among children with ADHD (Murias et al., 2007; Poil et al., 2014; Deiber et al., 2020). In contrast, other studies reported a reduction of alpha activity in children with ADHD interpreted as cortical hyperactivation or impaired attentional control (Loo and Makeig, 2012; Barry et al., 2003). We contextualize our findings within a recent study demonstrating electrophysiological heterogeneity across ADHD populations. Deiber et al. (2020) reported electrophysiological heterogeneity within ADHD populations and suggested the existence of distinct alpha-related profiles. Our study did not differentiate between alpha subtypes or electrophysiological phenotypes; the elevation of frontal alpha rhythm reflects broader neurophysiological variability rather than any single explanatory mechanism, such as delayed cortical maturation. The complexity and inconsistency of EEG data in the ADHD population require cautious interpretation of the resting-state alpha rhythm.

Although some condition-specific comparisons yielded statistically significant results (Tables 3, 5), these effects were isolated and inconsistent across measures and regions. Given the number of comparisons conducted and the absence of correction for multiple testing, these findings should be interpreted as exploratory. Accordingly, they do not substantially modify the overall pattern of largely stable EEG measures across assessment points.

The absence of EEG alterations may also suggest that resting-state EEG measures are relatively unchanged across a 3-month period in this age group. It is also possible that different neurophysiological markers (e.g., task-related ERPs or connectivity measures) may be more sensitive to intervention-related changes. Future studies incorporating multimodal and longitudinal approaches may help clarify these aspects. A follow-up EEG study may determine whether 3 months of cognitive training can cause significant changes in ADHD biomarkers. In particular, to determine the correlations between improved behavioral/cognitive aspects and EEG markers. More research is required to establish objective and definitive biomarkers of different subtypes of ADHD. The identification of distinctive EEG profiles may serve as a cost-effective biomarker and has prognostic value for accurate ADHD diagnosis and/or treatment. Future studies suggest conducting longitudinal research to track changes in brain connectivity and ADHD over time. Exploring treatment/intervention options to target individual symptoms in different subtypes may be useful (Li et al., 2025; The MTA Cooperative Group, 1999). We suggest that the greatest success in the treatment, management, and assessment of ADHD can be achieved through an integrated approach combining pharmacotherapy with cognitive and behavioral interventions.

The use of resting-state EEG in the present study was an intentional methodological choice, as we intended to examine condition-related spectral characteristics reflecting baseline neural organization rather than task-evoked neural responses. Resting EEG provides measures of intrinsic cortical activity. It allows assessment of state-dependent modulation (e.g., eyes-closed vs. eyes-open vs. eyes-closed), which are relevant for characterizing patterns of neural activity in children with ADHD (Bellato et al., 2020). While task-based EEG measures, such as event-related potentials, can provide complementary information about neural processes during specific cognitive demands (Johnstone et al., 2013). Resting EEG and spectral measures have been widely used in ADHD research to describe atypical oscillatory dynamics, including elevated slow-frequency activity and variations in the theta/beta ratio, across clinical and developmental samples (Loo and Makeig, 2012; Barry et al., 2003). Resting EEG has also been applied in longitudinal intervention studies. For example, Luo et al. (2023) employed resting EEG measures alongside cognitive and neurofeedback training, demonstrating the utility of baseline electrophysiological indices in intervention research. Similarly, pre-post resting EEG spectral changes have been reported in studies examining neurofeedback and other training paradigms in ADHD populations (Arns et al., 2014). Thus, resting-state EEG remains a valid and informative tool for characterizing baseline neural dynamics and their temporal modulation.

The present study primarily included children with the predominantly inattentive presentation of ADHD and excluded participants with significant psychiatric comorbidities and excessive movement during EEG recording. Excessive movement is known to introduce artifacts that compromise EEG signal quality and reduce data reliability. These methodological considerations were necessary to ensure valid neurophysiological measurement. Future studies including more heterogeneous clinical samples may help determine the extent to which these findings generalize to broader ADHD populations, which are more likely to be preliminary and potentially more reflective of children with predominantly inattentive presentations and relatively stable behavioral regulation during assessment. Further research with larger, more diverse samples and replication across different settings would contribute to drawing conclusions the broader generalizability of the results.

The study has some of limitations:

(1) The notable limitation is the absence of a control group, as EEG measurements were not collected from typically developing children. The inclusion of an age-matched control group would allow for more accurate comparison. It would help determine whether the lack of observed EEG changes is specific to children with ADHD or reflects general developmental or methodological factors.
(2) Relatively short duration of cognitive intervention and retesting period. EEG assessment was performed several days after the cognitive intervention. A 3-month intervention period may be insufficient to produce stable or long-term neurophysiological changes detectable on EEG. Future studies involving longer follow-up periods—assessments conducted 1-year after post-intervention—might be more informative for the identification and detection of EEG changes to assess the effects of cognitive intervention.
(3) There is an unequal number of participants of different age and gender groups. In addition, our sample was relatively homogeneous, consisting predominantly of children with the inattentive ADHD subtype and excluding those with comorbid conditions. Such disparity did not allow us to compare specific characteristics different age and gender groups in children with ADHD, and also limits the generalizability of our results.
(4) The limitation is associated with a lack of correlational analysis between EEG and cognitive intervention data. Results of the cognitive intervention and EEG data are being published separately due to the vast amount of data. As only preliminary EEG data analysis is presented here, a detailed correlational analysis, including all the results, is planned for publication in the future.
(5) The limitation concerns the comparison between included and excluded participants. Although Table 1 presents demographic characteristics for these groups, detailed clinical variables, such as symptom severity and baseline functional measures, were not available for this comparison. Consequently, potential differences in clinical presentation cannot be fully excluded, which may limit the representativeness of the analyzed sample.

## 5 Conclusion

Two possible explanations may account for the absence of observable changes in the EEG profile of children with ADHD, characterized by elevated low-frequency power, which was accompanied by elevated TBR across frontal, central, and parietal regions. First, a 3-month observation period may have been insufficient to detect measurable alterations in resting-state EEG. Second, ongoing neurodevelopmental processes may contribute to variability in cortical organization, potentially obscuring subtle temporal changes in EEG measures. Additionally, the observed elevation of frontal alpha activity may reflect broader neurophysiological variability and heterogeneity rather than delayed cortical maturation alone. Future research may help clarify the neurobiological links between longitudinal EEG dynamics and functional developmental trajectories, thereby contributing to the identification of potential neurophysiological biomarkers in children with ADHD.

## Data Availability

The datasets recorded and analyzed during the current study are available in the EEGHub repository (https://eeghub.ge/). The data are publicly accessible.
